# Probing the Cyanobacterial *Microcystis* Gas Vesicles after Static Pressure Treatment: A Potential In Situ Rapid Method

**DOI:** 10.3390/s20154170

**Published:** 2020-07-27

**Authors:** Jiajin Li, Ran Liao, Yi Tao, Zepeng Zhuo, Zhidi Liu, Hanbo Deng, Hui Ma

**Affiliations:** 1 Department of Biomedical Engineering, Tsinghua University, Beijing 100084, China; ljj18@mails.tsinghua.edu.cn (J.L.); liuzd17@mails.tsinghua.edu.cn (Z.L.); dhb19@mails.tsinghua.edu.cn (H.D.); 2 Division of Ocean Science and Technology, Shenzhen International Graduate School, Tsinghua University, Shenzhen 518055, China; 3 Guangdong Provincial Engineering Research Center for Urban Water Recycling and Environmental Safety, Shenzhen International Graduate School, Tsinghua University, Shenzhen 518055, China; tao.yi@sz.tsinghua.edu.cn; 4 Department of Physics, Tsinghua University, Beijing 100084, China; zzp17@mails.tsinghua.edu.cn (Z.Z.); mahui@tsinghua.edu.cn (H.M.)

**Keywords:** cyanobacterial blooms, gas vesicles, polarized light scattering, in situ detection, vertical migration

## Abstract

The vertical migration trend of cyanobacterial cells with gas vesicles in water ecosystems can reflect the changes in the natural environment, such as temperature, nutrients, light conditions, etc. The static pressure treatment is one of the most important approaches to study the properties of the cyanobacterial cell and its gas vesicles. In this paper, a polarized light scattering method is used to probe the collapse and regeneration of the cyanobacterial gas vesicles exposed to different static pressures. During the course, both the axenic and wild type strain of cyanobacterial *Microcystis* were first treated with different static pressures and then recovered on the normal light conditions. Combining the observation of transmission electron microscopy and floating-sinking photos, the results showed that the collapse and regeneration of the cyanobacterial gas vesicles exposed to different static pressures can be characterized by the polarization parameters. The turbidity as a traditional indicator of gas vesicles but subjected to the concentration of the sample was also measured and found to be correlated with the polarization parameters. More analysis indicated that the polarization parameters are more sensitive and characteristic. The polarized light scattering method can be used to probe the cyanobacterial gas vesicles exposed to different static pressures, which has the potential to provide an in situ rapid and damage-free monitoring tool for observing the vertical migration of cyanobacterial cells and forecasting cyanobacterial blooms.

## 1. Introduction

Cyanobacteria are one of the oldest organisms on earth which have had major impacts on shaping our modern-day biosphere [1,2]. However, the overgrowth of cyanobacteria frequently forms harmful algal blooms in the natural environment, which is posing ever-growing threats to both the aquatic ecosystem and human beings [3,4]. The growth and expansion of harmful cyanobacterial blooms are difficult to measure as affected by many environmental changes including phosphorus and nitrogen availability [5,6], climate change, water temperature raising [7,8], pH, and day length [9], etc. Moreover, like *Microcystis*, many planktonic cyanobacteria have the essential structure of gas vesicles that can regulate their buoyancy [10], so that they can easily migrate vertically in the water column [11] and form dense blooms in hours, in freshwater, estuarine, and marine ecosystems [3,12]. Therefore, in situ monitoring of the vertical migration of cyanobacterial cells can help us to forecast cyanobacterial blooms in the natural environment.

It is an emerging issue to observe the harmful cyanobacterial behaviors in situ. Scientists adopted the autonomous underwater vehicle that was equipped with many sensors to repeatedly pass through the phytoplankton layer and in situ monitor and track the marine ecological processes so as to deepen the understanding of distribution characteristics of harmful algae [13]. A toolbox was developed to routinely acquire the benthic sample and monitor the concentration of the secondary metabolites of cyanobacteria, making it possible to evaluate the risk of benthic cyanobacterial species [14]. Emerging technologies for the early detection of cyanobacterial blooms include fluorescence probes for real-time remote cyanobacteria monitoring [15,16], remote sensing detection [17], models based on driving factors like water temperature, pH, light, and so on to predict blooms [18]. These techniques have low sensitivity to the vertical migration observation, and can be susceptible to interferences by other water quality parameters or may be cost-prohibitive. Hitherto, the vertical migration of cyanobacterial cells is still difficult to observe in situ.

The gas vesicles formed solely from protein are inert, hollow, and gas-filled structures [19]. The buoyancy provided by the gas vesicles enables cyanobacteria to float upwards spontaneously and gather in the light area for a long time to obtain the opportunity for growth and proliferation, thus causing cyanobacterial blooms [20]. As a result, it is of great significance to monitor the state changes of the gas vesicles in situ, so as to observe the vertical migration of cyanobacterial cells and predict cyanobacterial blooms. The changes of cyanobacterial gas vesicles can be observed by using electron microscopy [21], capillary compression tube [22], turbidity [23], flow cytometry [24], and pressure nephelometry [25]. However, these methods are generally difficult to monitor the changes of gas vesicles in situ because of the sample preparations and ex situ measurements.

The static pressure treatment is one of the most important approaches to study the properties of the cyanobacterial cell and its gas vesicles, during which the cyanobacterial cells are exposed to the pressure exerted by a liquid or gas at rest. The static pressure treatment usually has the advantage of only destroying the gas vesicles of the cyanobacterial cell but preserving the intracellular organic matters [26,27]. The pressure of gas vesicles and its relationships with other intracellular parts have been investigated to better understand the diurnal migration of cyanobacteria [26]. The scattering properties of cyanobacterial *Microcystis* exposed to different static pressures were described in detail to deeply understand the optical characteristics of gas vesicles [28]. The effective critical collapse pressure of the gas vesicles in the cell, as an inherent property generally related to the metabolism of the cell, is measured after the static pressure treatment [25]. The *Microcystis* cells treated by static pressure have been used to study the relationship between the sinking *Microcystis* and the nitrate removal at the sediment-water interface [27]. Additionally, the static pressure treatment helps to investigate the impact of gas vesicles on the inherent optical properties of the cyanobacterial cell, which finally contributes to the retrieval of distinctive information concerning the water body from the satellite remote sensing data [29].

Polarization as the inherent property of light is sensitive to the changes of the microstructure of particles [30]. Parameters given by the polarized light scattering can provide more information to identify and classify the complex microstructure [31], and have been used to characterize the suspended particles in the seawater [32], classify the marine microalgae [33], or characterize the physiological states of the suspended marine microalgae [34]. Importantly, the polarization measurement sensor has been successfully instrumented to the in situ underwater instrument [35]. Our previous work has proven that the polarization parameters can be an effective evaluation method for the damage of the cyanobacterial cells when employing the sonication in the field blooms control [36,37].

Different static pressures help to control the changes of cyanobacterial gas vesicles in the cell, which enables us to find the polarization parameters sensitive to the changes of gas vesicles. In this paper, the changes of the polarization parameters of cyanobacterial gas vesicles obtained by exposing them to different static pressures were investigated, so as to propose a method for the vertical migration observation in the water column. The results showed that the collapse and regeneration of *Microcystis* gas vesicles can be characterized by the polarization parameters. TEM images and floating-sinking photos were given to indicate the state changes of *Microcystis* gas vesicles. The turbidity was measured and compared with the polarization parameters which are more sensitive and characteristic. It is expected to provide a rapid and damage-free method for in situ probing of the cyanobacterial gas vesicles and foreseeing the vertical migration trend.

## 2. Materials and Methods

### 2.1. Microorganisms

Axenic *Microcystis aeruginosa* (No. FACHB 905, Freshwater Algae Culture Collection of the Institute of Hydrobiology, Chinese Academy of Sciences, Wuhan, China), which is the most frequently reported cyanobacterial species for blooming and toxicity in Chinese waters. These axenic *M. aeruginosa* cells were cultured and sampled five times for the static pressure treatment and measurements.

The wild type strain of *Microcystis* (WT) was collected three times during *Microcystis* blooms in August 2019, from the surface of Lake Taihu (31°43′ N, 120°23′ E).

### 2.2. Experimental Setup for Polarization Measurement

**Figure 1:**
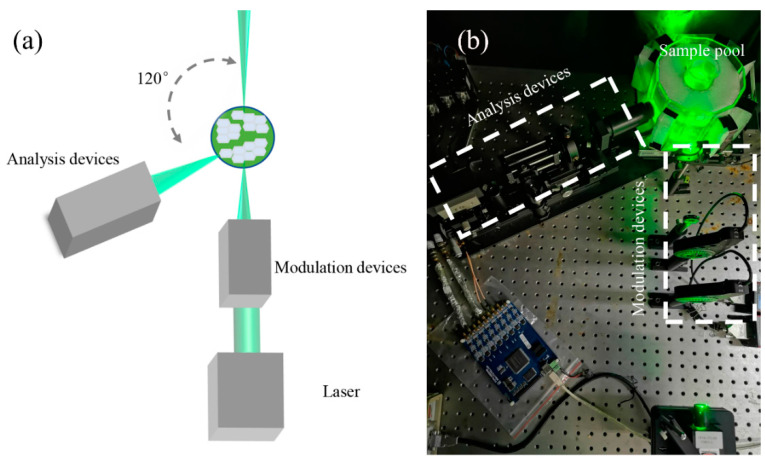
The experimental setup for polarization measurement (**a**) The schematic diagram. (**b**) The physical diagram.

Based on our previous report [38], an experimental setup was designed to measure the scattered polarization parameters of the suspended cyanobacterial cells as individual particles, as shown in Figure 1a,b.

The polarized light generated by modulation devices illuminates the individual suspended particle in the sample pool and then scatters. After that, the analysis devices will collect the scattered light as pulses and simultaneously measure the corresponding Stokes vectors. The scattering volume is reduced to less than 0.01 μL. When the particle concentration is less than 10^5^ particles per mL, there should be only one particle on average in the scattering volume and the individual particle measurement can be achieved [38]. Therefore, if the concentration is below 10^5^/mL, a single free cell or cell aggregate of the cyanobacteria (as a particle) is individually measured and less affected by the concentration. For each measurement in this study, we recorded more than 3000 pulses each sample by the setup and then obtained the Stokes vectors with the same numbers, which enabled us to get the stable distributions of the polarization parameters.

### 2.3. Analytical Methods

The Stokes vector S as defined in Equation (1) can be used to represent the polarization state of light.

(1) $$\;\;\;\;S=\left[\begin{array}{c} I\\ Q\\ U\\ V \end{array}\right],$$

where I is the total intensity, Q and U respectively are the residual polarized intensities on the 0° and 45° linearly-polarized directions, and V is the residual right-circularly polarized intensity.

The elements of S normalized by the upper limit of the intensity are dimensionless parameters in the following context. The q, u, and v can be defined by Equation (2) only to consider the polarization part of S [38]. Therefore, I is in the range from 0 to 1, and q, u, and v are in the range from −1 to 1. Further, the [I, q, u, v ] are changed into [|I|, |q|, |u|, |v| ] to make all elements fall within the range from 0 to 1.

(2) $$\;\;\;\;q=\frac{Q}{I},u=\frac{U}{I},v=\frac{V}{I}.$$

The pulse data of two different samples can be obtained with the same sampling number by our experimental setup, and further, two data sets of [|I|, |q|, |u|, |v|] which are the multidimensional polarization parameters can be calculated using the pulse data [38]. Linear discriminant analysis (LDA) is a popular dimensionality reduction technique to solve the problem of information fusion and data mining of two data sets in higher dimension space [39]. LDA can find the optimal linear combination of |I|, |q|, |u|, and |v| which maximizes the target function M as defined in Equation (3) to project two data sets from high dimensional space to one-dimensional space [38,40].

(3) $$M=\frac{{\left|{µ}_{1}-{µ}_{2}\right|}^{2}}{\omega_{1}+\omega_{2}}$$

where ${µ}_{1}$ and ${µ}_{2}$ are the means of two data sets after projection, while $\omega_{1}$ and $\omega_{2}$ are the variances of two data sets after projection.

Herein, for simplicity and intuition, two data sets of [|I|, |q|, |u|, |v|] of cyanobacterial cells with and without gas vesicles are trained using LDA to find the maximal *M* and meantime obtain the optimal linear combination, that is, the one-dimensional polarization parameter x as defined in Equation (4). Then, the original data sets are transformed into one-dimensional distributions of the polarization parameter x which are the best separation of the cyanobacterial cells with and without gas vesicles.

(4) x=f([|I|,|q|,|u|,|v|]).

where R can be defined as shown in Equation (5) to accurately quantify the separation of two distributions of the x of the algae cells in two different states (control cells and treated cells).

(5) $$R=1-\ln\left(\frac{2*P}{F_{1}+F_{2}}+1\right),$$

where P is the distance between the peaks of two distributions, which is representative of the difference between classes (having gas vesicles or not); $F_{1}$ and $F_{2}$ are the full widths at half maxima of two distributions, which are representative of the deviations within each class. Accordingly, two distributions are similar while the *R*-value is close to 1.

### 2.4. The Pressurization Device

**Figure 2:**
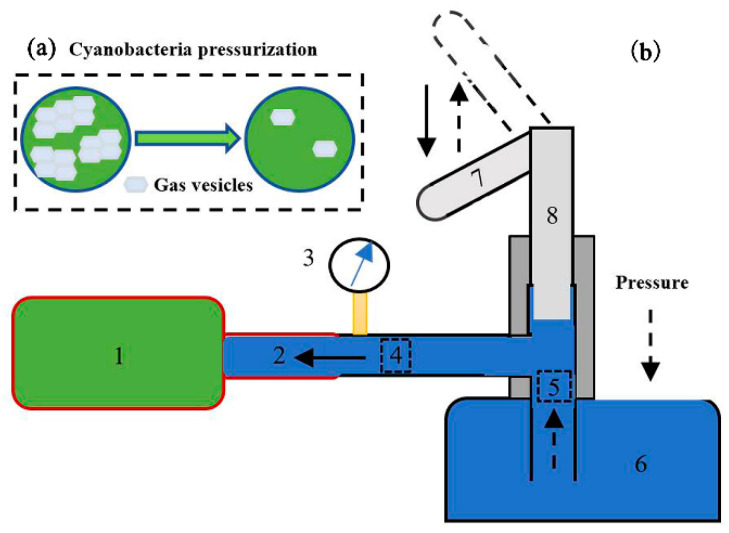
(**a**) The cartoon pattern for the change of cyanobacterial cells after pressurization. (**b**) The schematic diagram of the pressurization device. 1, sample bottle; 2, threaded joint with sealing ring; 3, manometer; 4, check valve A; 5, check valve B; 6, water tank; 7, handle; 8, plunger.

Cyanobacterial cells can be pressurized in different static pressures in the pressurization device as shown in Figure 2. The sample bottle (1#) filled with algae cells is connected to a threaded joint with a sealing ring (2#). When handle (7#) is lifted, distilled water in the water tank (6#) enters into the pipeline (between check valve A and check valve B) through check valve B under atmospheric pressure. When the handle is pressed down, the distilled water in the pipeline will be squeezed and passes through the check valve A to exert water pressure to the sample bottle. The pressure in the sample bottle can be accumulated by repeating this procedure to the desired pressure values which are shown and read by the manometer (3#).

For cyanobacterial cells with gas vesicles, the status of gas vesicles will significantly change after pressurization, as shown in Figure 2a. Studies have shown that when *Microcystis* cells are subjected to pressure larger than 0.7 MPa, all their gas vesicles will collapse [27]. To study the variations of *Microcystis* gas vesicles, different static pressures of 0–0.7 MPa were exerted to the *Microcystis* cells.

## 3. Results

### 3.1. Effectiveness of Polarized Light Scattering Method on Monitoring the Collapse of Gas Vesicles of Microcystis Cells after Static Pressure Treatments

Static pressure gradients of 0.1, 0.3, 0.4, 0.5, 0.6, and 0.7 MPa were set and compared with the control group (0 MPa). The *M. aeruginosa* sample was mixed and poured into seven centrifuge tubes. For the sample in each tube, we transferred them to the sample bottle of the pressurization device (Figure 2) and operated it to reach the desired static pressure which was read from the manometer and maintained for 5 min. After that, we released the sample bottle and transferred the sample back to its centrifugal tube. After the static pressure treatment, the sample in each centrifugal tube was shaken and mixed, and taken into the sample pool to measure the scattered polarization parameters. The same procedure was performed on the WT sample.

#### 3.1.1. The Distributions of the Polarization Parameter and *R*-Value of *Microcystis* Cells after Static Pressure Treatments

It was known that all gas vesicles in *Microcystis* cells will collapse under 0.7 MPa static pressure [27]. In order to find the coefficients most strongly dependent on the gas vesicle volume, the data of the control group and 0.7 MPa group were used as the two data sets of LDA. And then the polarization parameter x was found that maximize M in Equation (3) between the two data sets. In this way, the polarization parameters, $x_{1}$ and $x_{2}$, were obtained for *M. aeruginosa* and WT samples respectively. For easily comparing, we shared the formula $x_{1}$ and transferred the scattered polarization parameters data of other *M. aeruginosa* pressures groups to $x_{1}\;$, and the same procedure was performed on WT samples to find $x_{2}$ and transfer the data.

**Figure 3:**
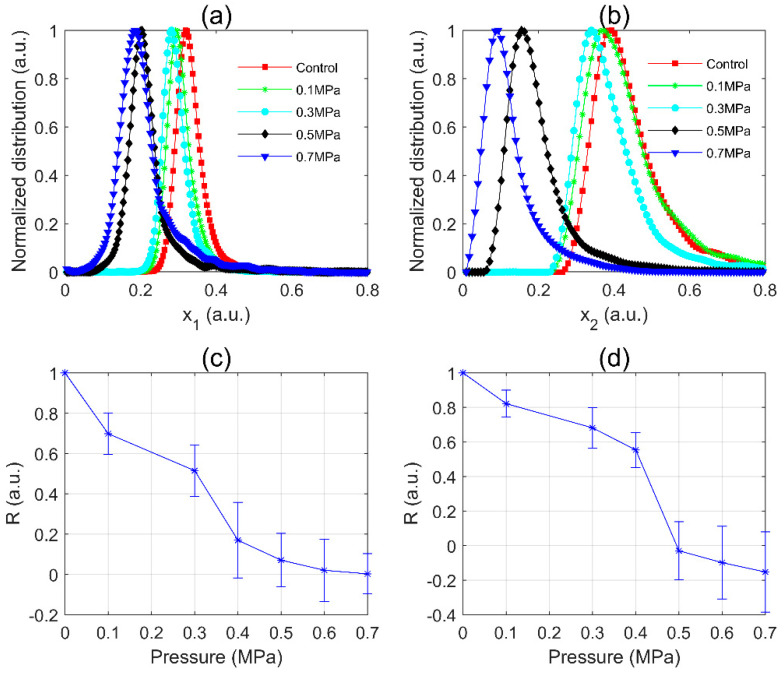
The distributions of the polarization parameter and *R*-value of (**a**,**c**) *M. aeruginosa*; (**b**,**d**) WT of different static pressure treatments.

The normalized distributions of *M. aeruginosa* are shown in Figure 3a. The distances between each peak of pressurized samples and that of the control group, the *P*-value, became larger with the higher treatment pressure. It suggested that the *P*-value is sensitive to the change of static pressure. For the WT samples as shown in Figure 3b, the *P*-value similarly became larger with the higher treatment pressure. However, the width of the distribution of *M. aeruginosa* 0.7 MPa group was wider than that of the control group; while for WT, the width of the distribution of 0.7 MPa group was significantly narrower than that of its control group.

Further, the *R*-values of both *M. aeruginosa* and WT were calculated as shown in Figure 3c,d, respectively. The distributions in Figure 3a,b were stable since each of their data sets included at least 3000 samplings of cells in each sample. Therefore, *R*-values calculated by Equation (5) were reliable. And considering the variance of different samples, we conducted similar experiments for at least three times. As the static pressure increased, the *R*-values of both *M. aeruginosa* and WT samples decreased monotonically, and the decline of *R*-value with the pressure was evident and impressive. In Figure 3c, the slope of *R*-value of *M. aeruginosa* within 0.1–0.3 MPa fell gently and the downward trend was the most significant within 0.3–0.4 MPa, then flattened out after 0.5 MPa. In Figure 3d, the slope of *R*-value of WT within 0.1–0.4 MPa fell gently and became steeper within 0.4–0.5 MPa. The results indicated that the *R*-value was positively correlated with the static pressure exerted on the *Microcystis* cells. Moreover, there was a critical pressure that caused the *R*-value of *Microcystis* to change sharply. By careful comparisons, the *R*-value in Figure 3d changes sharper than that of Figure 3c after critical pressure, because the wild type strain of *Microcystis* has a greater volume ratio of the gas vesicles in the cells than those of the axenic *M. aeruginosa* [3,41,42].

#### 3.1.2. TEM Images of WT after Different Static Pressure Treatments

**Figure 4:**
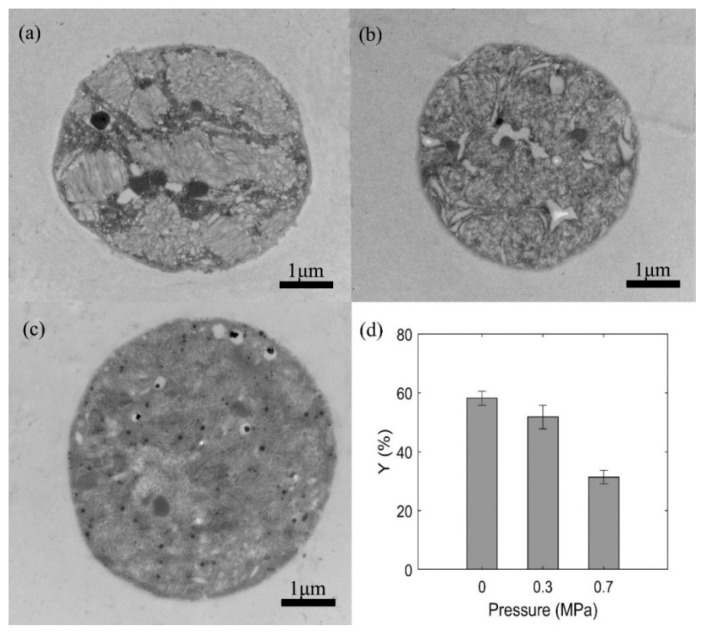
TEM images of WT (**a**) without static pressure treatment, (**b**) pressurized by 0.3 MPa, (**c**) pressurized by 0.7 MPa, and (**d**) the white pixel percentage of intracellular gas vesicles (*Y*-value) using the binarization analysis.

Transmission electron microscopy (TEM) was used to observe the intracellular structure of WT shown as Figure 4a,c. The samples pressurized by 0, 0.3, 0.7 MPa were immediately taken and fixed after pressure treatments to conduct the TEM observation. And the pre-treatment procedure of *Microcystis* cells for TEM observation had no significant effects on the gas vesicles [19,42]. The gas vesicles of the control sample almost filled with the cell and were arranged in a regular pattern (Figure 4a). However, for the sample pressured at 0.3 MPa (Figure 4b), the gas vesicles were scattered and reduced in number although there were still residual gas vesicles inside the cell. For the sample pressured at 0.7 MPa (Figure 4c), there were no visible gas vesicles inside the cell. Besides, it showed that the static pressure treatment did not damage other structures of the *Microcystis* cell.

As the gas vesicles were much whiter than the other parts of the cell in TEM images, the TEM images were binarized and the proportion of white pixels were calculated to represent the sectional area ratio of gas vesicles to the cell, named as *Y*-value, shown in Figure 4d. The results indicated that the gas vesicles were significantly reduced after the static pressure treatments. The *Y*-values of samples exposed to 0.3 and 0.7 MPa reduced to 89% and 54% of the control sample, suggesting that such reduction trends were positively correlated with pressure. It should be noticed that the *Y*-value of 0.7 MPa group was still larger than 20%, possibly due to the other intracellular organelles contributed to the white pixels.

The collapse of gas vesicles would lead to the optical and physical changes of the cells, which can be indicated by the scattered light. Previous reports indicated that the results of TEM images and turbidity were dependent on the pressure value [42], and the disappearance of gas-filled spaces by static pressure treatment would result in a lower difference of the refractive index with the surrounding aqueous medium, which can cause a decrease in turbidity [23,26].

#### 3.1.3. Settlement Performances of WT after Static Pressure Treatments

**Figure 5:**
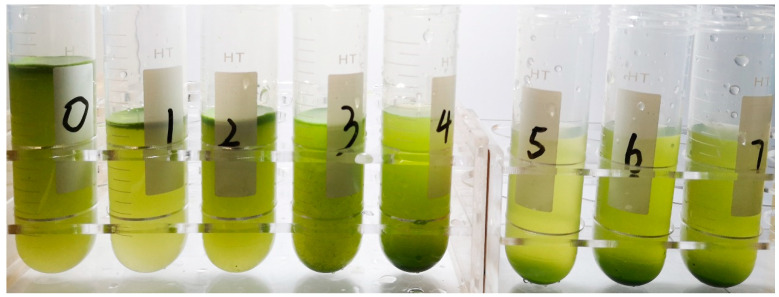
The settlement performances of WT cells in the water column after different static pressure treatments. The tubes from left to right were the samples pressurized by 0, 0.1, 0.2, 0.3, 0.4, 0.5, 0.6 and 0.7 MPa, respectively.

The WT samples after static pressure treatments were placed in centrifuge tubes for observing the settlement performances, as shown in Figure 5. The samples exposed to 0.1–0.2 MPa pressure were similar to the control sample, and most of the cells floated on the water surface. For the samples exposed to 0.3 MPa pressure, most cells turned to suspended state and part of cells settling to the bottom. However, for cells pressurized by 0.4 MPa, no obvious surface layer existed, but the majority of cells suspended in the lower layer close to the bottom. When pressurized by 0.5–0.7 MPa, almost all cells sunk to the bottom. It confirmed that the static pressure treatment led the gas vesicles of WT cells to collapse, as the cells lost buoyancy and sunk downward.

After the pressure increased to 0.5 MPa and above, the proportions of the cells at the bottom increased, while few cells were visible on the surface. Therefore, the pressure of 0.4–0.5 MPa can be considered as the critical pressure that caused WT cells to lose gas vesicles significantly and settle downward. Recalling the *R*-value shown in Figure 3d, when the pressure reached 0.5 MPa, the *R*-value dropped sharply, which confirmed that the *R*-value can be adopted as an indicator of the settling state of WT cells.

### 3.2. Effectiveness of Polarized Light Scattering Method on Monitoring Regeneration of Gas Vesicles of Microcystis Cells during Post-Pressurization Incubation

After static pressure treatments, the *M. aeruginosa* samples were cultured for 72 h, respectively, at 25 °C temperature and 1500 lx irradiance all the time, without stirring. At 0, 12, 24, 36, 48, and 72 h, the polarization parameters of samples were measured. The TEM observation and settlement performance observation were conducted synchronously. The same procedure was performed on WT samples. In order to better compare the changes of gas vesicles in the cell, the *x_1_* and *x_2_* which were mentioned in Section 3.1.1 were still used to transfer the data of *Microcystis* cells during post-pressurization incubation.

#### 3.2.1. The Distributions of Polarization Parameter and R-Value of *Microcystis* Cells during Post-Pressurization Incubation

**Figure 6:**
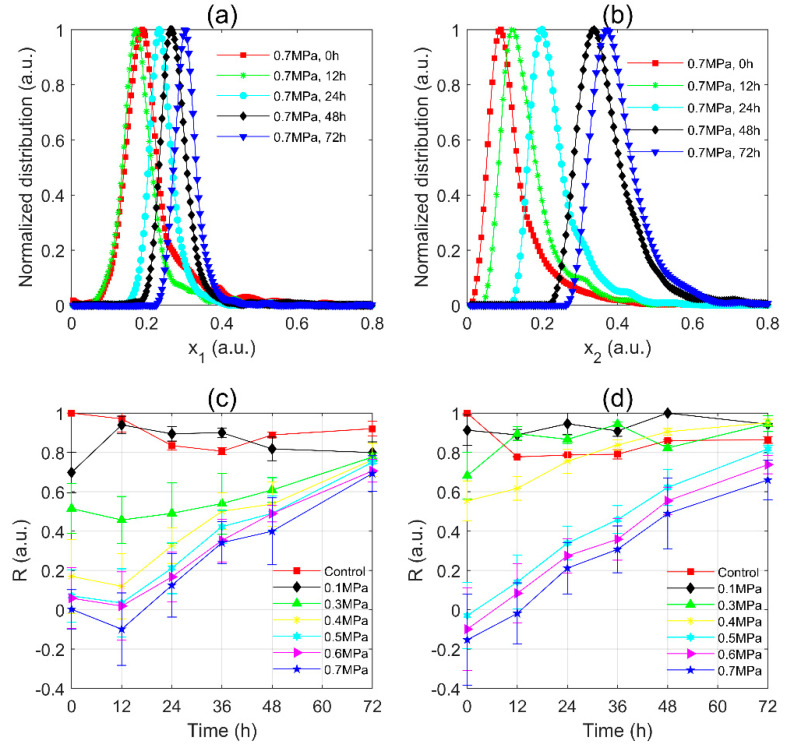
The distributions of polarization parameter and *R*-values of (**a**,**c**) *M. aeruginosa*; (**b**,**d**) WT during post-pressurization incubation.

The distributions of *M. aeruginosa* samples during post-pressurization incubation were shown in Figure 6a. The distribution at 12 h moved to the left slightly (smaller $x_{1}$) and then moved to the right monotonically (larger $x_{1}$). Meanwhile, the width of the distribution was narrowed and reached its minimum at 72 h. It noted that the peak and width of the distribution were finally close to the control group. Similarly, the results of the WT were shown in Figure 6b. The distribution moved to the right monotonically. However, in contrast to Figure 6a, the width of the distribution became wider with culture times.

The *R*-values were calculated and shown in Figure 6c for *M. aeruginosa* and Figure 6d for WT samples. In Figure 6c, the *R*-values of the control group ranged from 0.8 to 1, similar to those of 0.1 MPa group. The *R*-values of 0.3–0.7 MPa groups slightly decreased at 12 h, and then monotonically grew up to approach the value of the control group. In Figure 6d, the *R*-values of 0.1 MPa group and 0.3 MPa group fluctuated in the range of 0.8–1 as the culture time increased, which cannot differentiate from those of the control group. The *R*-values of 0.4 MPa group increased with the culture time and after 24 h, they fell into the fluctuation range of the control group which meant that the sample recovered to a similar state as the initial sample. Similarly, the *R*-values of 0.5–0.7 MPa group monotonically grew as the culture time increased, but were diverse at 72 h and the differences between them were much larger than those in Figure 6c. It noted that after 72 h regeneration, the *R*-value of 0.7 MPa group was still quite smaller than the control group. Similar to those in Figure 3, the distributions and the calculated *R*-values were based on at least 3000 samplings of the cells in each sample, which ensured the reliability of the distributions and *R*-values. Multiple experiments were conducted with different samples, which were shown in Figure 6c,d. One can see that, although there are some overlaps due to the error bars, the tendency of *R*-value with the culture times was evident.

#### 3.2.2. TEM Images of WT during Post-Pressurization Incubation

**Figure 7:**
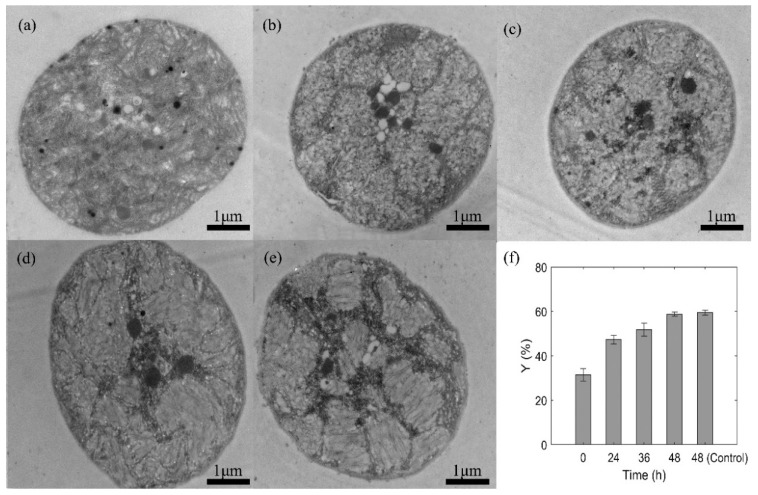
TEM images of WT pressurized by 0.7 MPa at (**a**) 0 h; (**b**) 24 h; (**c**) 36 h; (**d**) 48 h; (**e**) Control group at 48 h. (**f**) the *Y*-value changed as the culture time.

The samples pressurized by 0.7 MPa at 0, 24, 36, and 48 h, and the sample of the control group at 48 h were taken and fixed to conduct the TEM observation. The TEM images of WT pressurized by 0.7 MPa during post-pressurization incubation were shown in Figure 7. There were no visible gas vesicles inside the cells after pressurization at 0 h (Figure 7a). As the culture time increased, the gas vesicles reappeared but were irregular at 24 h and 36 h (Figure 7b,c), and then became dominated at 48 h, (Figure 7d), which was similar to the control group (Figure 7e). However, by carefully comparing Figure 7d,e, the shape of gas vesicles in the recovered cell was less aligned than the control group.

The *Y*-values of TEM images were calculated and shown in Figure 7f. As the culture time increased, the *Y*-values increased and approached to that of the control group.

#### 3.2.3. Settlement Performances of WT during Post-Pressurization Incubation

**Figure 8:**
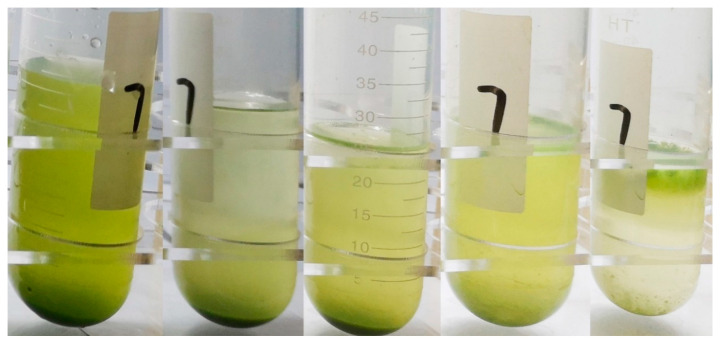
The settlement performances of WT cells in the water column during post-pressurization incubation (from left to right, 0, 24, 48, 72, 96 h respectively).

The settlement performances of WT during post-pressurization incubation were shown in Figure 8. From left to right, there were WT pressurized by 0.7 MPa respectively at 0, 24, 48, 72, 96 h. The cells distributed at the bottom at 24 h and there were few cells in the water column. At 48 h, the suspension became turbid, but most of the cells still distributed at the bottom. At 72 h, most of the cells were distributed in the water column, and fewer cells stayed at the bottom. At 96 h, there was an obvious layer of cells floating on the surface. From Figure 7 and Figure 8, it should be noticed that the tiny structure difference between the recovered cell at 48 h and that in the control group would lead to quite different buoyance behavior.

## 4. Discussion

The traditional scattering-based optical instruments like turbidity and flow cytometry to detect the gas vesicles mainly depended on the data of the light intensity. The mentioned polarized light scattering method can not only obtain the data of light intensity but also obtain the data of the polarization part of the light. In the above results, the data of intensity and polarization part were combined as a whole to analyze, which can improve the recognition rate of cells in different states. However, the data of intensity and polarization part maybe have different responses on the process of the collapse and regeneration of gas vesicles. In the following discussion, the relationship between turbidity and polarization parameters is shown, and the superiorities of polarization parameters are present.

The turbidity of the algae suspension is measured using a Turbidity Meter (2100Q, HACH, Loveland, CO, USA). Turbidity Meter measures the ratio of the bulk light intensity of suspension scattered at the 90° scattering angle to the intensity of the illuminating light, and its value mainly depends on the concentration.

### 4.1. The Changes of Turbidity and the Relationship between R and Turbidity

**Figure 9:**
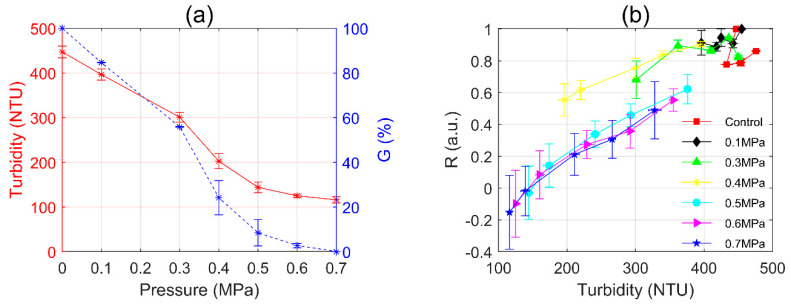
(**a**) The turbidity (left, red star-solid line) and the percentage of gas vesicles (right, blue star-dashed line) after different static pressure treatments; (**b**) The relationship between *R* and turbidity.

The turbidity measurement is dependent on the data of light intensity, and its value generally depends on the concentration. Figure 9a (red star-solid line) shows the turbidity of WT cells after different static pressure treatments. The turbidity of initial WT suspension was 447 NTU and significantly declined as the pressure increased, and finally reduced to 116 NTU after 0.7 MPa static pressure treatment.

The decrease in turbidity is proportional to the number of gas vesicles, so that the percentage of gas vesicles G can be calculated by Equation (6) [26].

(6) $$\;\;G=\frac{T_{b}-T_{c}}{T-T_{c}}\times 100\%,$$

where *T* is the initial turbidity of the suspension; $T_{b}$ is the turbidity remaining of the suspension after the static pressure treatment; $T_{c}$ is the turbidity remaining of the suspension after all the gas vesicles collapsed. Since the TEM images in Figure 4c show that the gas vesicles collapsed under 0.7 MPa for 5 min, here $T_{c}$ was measured after the WT suspension was treated under the same conditions.

In Figure 9a (blue star-dashed line), we calculated the *G*-values and got the percentage of gas vesicles after different static pressure treatments. After 0.3 MPa pressurization treatment, the percentage of gas vesicles of the WT still was 55.9% and it sharply declined to 24.2% at 0.4 MPa. After 0.5 MPa pressurization treatment, it reduced to 8.5%. According to Figure 5, when the percentage of gas vesicles remained 24.2%, most of the WT cells floated on the lower layer, and when the percentage of gas vesicles was less than 8.5%, the cells sunk to the bottom. Recalling Figure 3c,d, we can easily find that there is a correlation between *R*-value and turbidity.

We also measured the turbidity of the samples every 12 h during 48 h post-pressurization incubation, and correspondingly we plotted the relationship of *R*-turbidity shown in Figure 9b. The values of *R* and turbidity of control and 0.1 MPa group were in a small range of variation. In the 0.3–0.4 MPa group, the values of *R* and turbidity were slightly reduced but were finally close to the control group at 48 h. As pressure increased, in the 0.5–0.7 MPa group, the values of *R* and turbidity changed consistently and had a significant reduction compared to the control group.

Summarily, the relationship of *R*-turbidity is correlated but nonlinear, which indicated that *R* may contain more information than turbidity. It indicated that both the intensity and the polarization data were included in the evaluation of the *R*-value, while turbidity measurements only considered the intensity. Therefore, the information carried by the intensity and the polarization part should be demonstrated and compared.

### 4.2. Comparison of Light Intensity and Polarization Part

In order to know the contributions of intensity *I* and polarization parameters q, u, v , we divided x=f([|I|,|q|,|u|,|v|]) into x=|I| and the polarization part x=f([|q|,|u|,|v|]) . Because the polarization part was still multiple parameters, we also used LDA to respectively find the formula $x_{3}$ of *M. aeruginosa* and $x_{4}$ of WT which minimized the *R*-value of 0.7 MPa group at 0 h and transferred other data as described above at Section 3.1.1.

**Figure 10:**
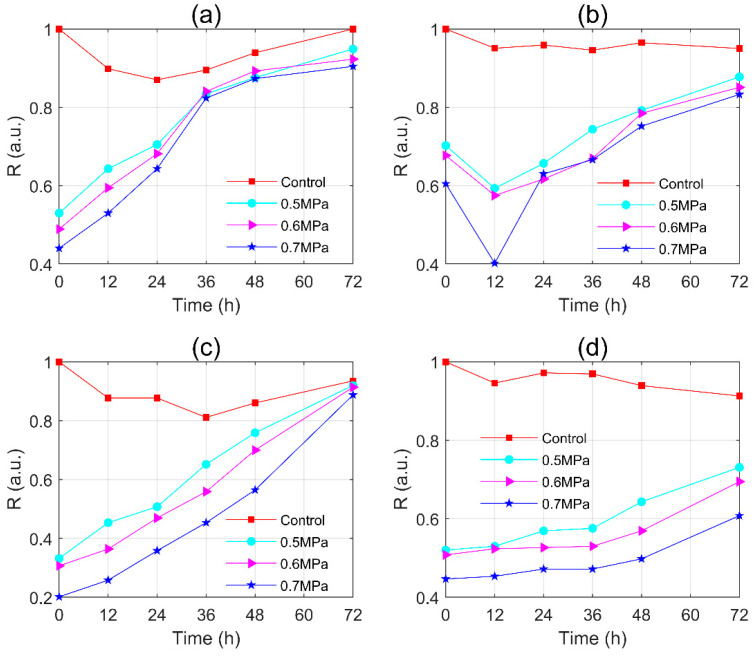
The *R*-values of x=|I| with culture times of (**a**) *M. aeruginosa*; (**c**) WT; The *R*-values of x=f([|q|,|u|,|v|]) with culture times of (**b**) *M. aeruginosa*; (**d**) WT.

The *R*-values of x=|I| both *M. aeruginosa* and WT were shown in Figure 10a,c. The *R*-values of the control group ranged from 0.8 to 1, and the *R*-values of 0.7 MPa group increased monotonically with the culture time and can be close to the control group at 72 h.

For *M. aeruginosa* samples, the *R*-values of $x_{3}$ of the control group and 0.5–0.7 MPa group were collected in Figure 10b. The *R*-values of the control group fluctuated in the range from 0.9 to 1. The *R*-values of 0.5, 0.6, and 0.7 MPa groups fell into a dip-like decreasing at 12 h which depended on the pressures. Then, the *R*-values continuously rose, but still had a certain difference with that of the control group at 72 h. The *R*-values’ difference between the different pressure groups were quite small after 72 h.

For WT samples, the *R*-values of $x_{4}$ were collected in Figure 10d. The *R*-values of the control group fluctuated in the range from 0.9 to 1. The *R*-values of 0.5, 0.6, and 0.7 MPa groups grew very slowing with the culture time before 36 h and then speeded up the rising rate, but they were far from that of the control group at 72 h. It can be noted that, in Figure 10d, the *R*-values of these pressure groups almost kept the difference between them at 0 h with the culture time increased, which was quite different from those in Figure 10b.

In Figure 10a,b, both |I| and $x_{3}$ can demonstrate the regeneration of the gas vesicles of *M. aeruginosa* samples. Relatively, $x_{3}$ can cover more time duration, since $x_{3}$ can work even after 36 h when *I* fell in the fluctuation range of that of the control group. In addition, there was a dip-like of $x_{3}$ at 12 h which indicated an intracellular structure change, but |I| had no response.

Similarly, from Figure 10c,d, it can be seen that both |I| and $x_{4}$ also can demonstrate the regeneration of the gas vesicles of WT samples, but $x_{4}$ still had much more potential than |I| to characterize the regeneration after 72 h. From Figure 8, one can find that the cells distributed in the water column at 72 h, and the majority of cells would not float at the surface until 96 h. The *R*-value of $x_{4}$ almost kept static before but changed significantly after 36 h, which indicated that the polarization part would be more characteristic of the buoyance changes of the cells than the intensity.

Traditional turbidity is a bulk measurement of the scattered intensity ratio and is subjected to the concentration of the suspensions, and more importantly, it ignores many differences of the particles because of the whole measurement [43]. However, our setup can individually measure the free cell or cell aggregation, and measure the scattered polarization parameters which include the intensity and polarization part [38]. Hence, it is promising its sensitivity to the microstructure of cells. The obtained distributions of the polarization parameters can carry more information about the sample and therefore can be more characteristic. The statistical magnitudes of the distributions, such as peak and full width at half maxima, were used to evaluate the *R*-value, which resulted in *R* being more direct and sensitive to exhibit the states’ change of the cyanobacterial gas vesicles than the turbidity.

Briefly, we can conclude that polarization is more sensitive to the changes of gas vesicles in the cell, and it can provide more specific points and more intracellular structure information for us to analyze the *Microcystis* cells.

### 4.3. The Micrograph of WT Sample

**Figure 11:**
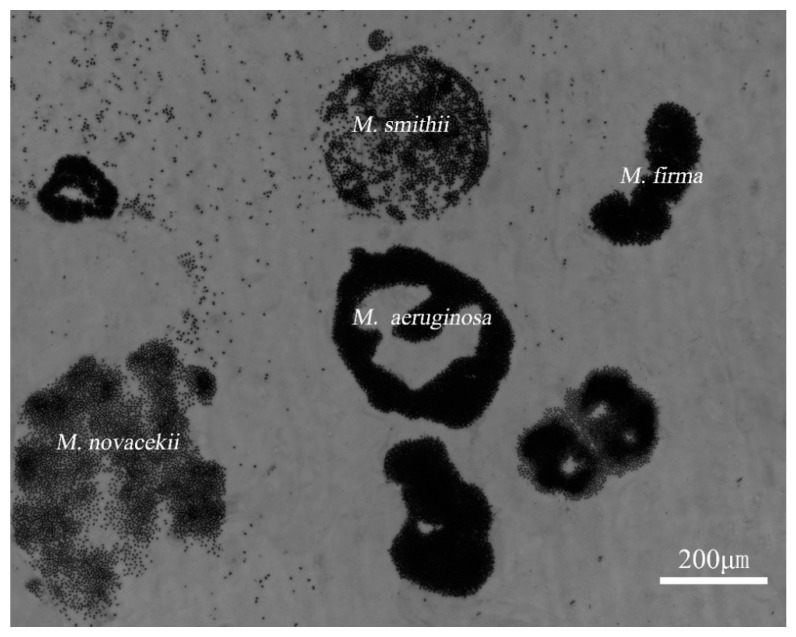
The micrograph of the WT sample.

We took the micrograph of the WT sample as shown in Figure 11 and found that the sample consisted of many kinds of *Microcystis* with gas vesicles, such as *M. aeruginosa*, *M. smithii, M. firma, M. novacekii* [44], which indicated that in situ measurement should find the more sensitive and specific parameter for the gas vesicles because of the complexity of particles. Notably, our setup individually measures the polarization parameters of the particle, which can provide multidimensional data and recognize the differences between different particles. The polarization parameters can characterize the gas vesicles in cells, as the common structure of *Microcystis*, and the given polarization parameters may have less relationship with the specific species of *Microcystis* with different shapes or other structures.

Importantly, these results implied that the gas vesicles may dominate the polarization parameters. Recalling Figure 6a,b, during the regeneration of the gas vesicles of *M. aeruginosa*, the widths of the polarization parameter distributions lessened with the culture time, but they enlarged for WT samples. Since the *M. aeruginosa* samples were lab-cultured and had almost similar gas vesicles, their widths of the polarization parameter distributions narrowed after the gas vesicles grew up. However, for WT samples, the gas vesicles were different because of the different species and different intracellular structures. When the gas vesicles collapsed, the difference from shapes or other structures cannot contribute to the wide distribution, so the width of distributions narrowed. However, when the gas vesicles recovered and they dominated the polarization properties of the samples, the widths of the polarization parameter distributions broadened.

If we consider the overall experimental results of the gradient pressurization experiments and regeneration experiments from Figure 3, Figure 6, and Figure 10, it can be summarized that the polarization parameters are strong and robust enough to specifically characterize the gas vesicles of cyanobacterial *Microcystis* beyond the species diversity.

## 5. Conclusions

The static pressure treatment is a proven effective approach that can break the cyanobacterial gas vesicles with little damage to other cellular structures. The polarized light scattering setup was used to measure the *Microcystis* samples exposed to different static pressures. The axenic *M. aeruginosa* and the wild type strain of *Microcystis* were sampled. The scattered polarization parameters of the suspended particles (single cell or individual cell aggregation) were individually measured by our setup, which is rapid and damage-free. Firstly, the gradient pressurization experiments and the supplemented experiments of TEM and floating-sinking photos indicated that it was the cyanobacterial gas vesicles dominating the changes of the polarization parameters. Moreover, the results of the regeneration experiments and the related supplemented experiments showed the polarization parameters can characterize and monitor the regeneration of the cyanobacterial gas vesicles. Discussions showed that the proposed polarization parameters were generally consistent with the measured turbidity when the *Microcystis* gas vesicles collapsed and regenerated. Further, more analysis showed that both the individually measured intensity and polarization part can characterize the changes of gas vesicles, but the polarization part can be more sensitive and work for a broader range. Furthermore, the micrograph of WT samples showed that the polarization parameters are strong and robust enough to characterize the gas vesicles of *Microcystis* beyond the species diversity. Also, the results of the regeneration of the gas vesicles showed that the polarization parameters were closely related to the vertical migration of the cyanobacterial cells, which implied that the future underwater instrument would help to forecast the cyanobacterial blooms. In summary, the polarized light scattering method and its polarization parameters can be expected to provide a rapid damage-free method for in situ probing of the cyanobacterial gas vesicles and the vertical migration observation, and thus they can be used in cyanobacterial bloom forecast and water environment treatment.
